# Early life determinants of low IQ at age 6 in children from the 2004 Pelotas Birth Cohort: a predictive approach

**DOI:** 10.1186/s12887-014-0308-1

**Published:** 2014-12-16

**Authors:** Fabio Alberto Camargo-Figuera, Aluísio JD Barros, Iná S Santos, Alicia Matijasevich, Fernando C Barros

**Affiliations:** Postgraduate Program in Epidemiology, Federal University of Pelotas, Pelotas, Brazil; Universidad Industrial de Santander (UIS), Bucaramanga, Colombia; Department of Preventive Medicine, School of Medicine, University of São Paulo, São Paulo, Brazil; Postgraduate Program in Health and Behavior, Catholic University of Pelotas, Pelotas, Brazil

**Keywords:** Child development, Birth cohort, Intelligence, Cognition, Social determinants of health, Brazil

## Abstract

**Background:**

Childhood intelligence is an important determinant of health outcomes in adulthood. The first years of life are critical to child development. This study aimed to identify early life (perinatal and during the first year of life) predictors of low cognitive performance at age 6.

**Methods:**

A birth cohort study started in the city of Pelotas, southern Brazil, in 2004 and children were followed from birth to age six. Information on a broad set of biological and social predictors was collected. Cognitive ability—the study outcome—was assessed using the Wechsler Intelligence Scale for Children (WISC). IQ scores were standardized into z-scores and low IQ defined as z < −1. We applied bootstrapping methods for internal validation with a multivariate logistic regression model and carried out external validation using a second study from the 1993 Pelotas Birth Cohort.

**Results:**

The proportion of children with IQ z-score < −1 was 16.9% (95% CI 15.6–18.1). The final model included the following early life variables: child’s gender; parents’ skin color; number of siblings; father’s and mother’s employment status; household income; maternal education; number of persons per room; duration of breastfeeding; height-for-age deficit; head circumference-for-age deficit; parental smoking during pregnancy; and maternal perception of the child’s health status. The area under the ROC curve for our final model was 0.8, with sensitivity of 72% and specificity of 74%. Similar results were found when testing external validation by using data from the 1993 Pelotas Birth Cohort.

**Conclusions:**

The study results suggest that a child’s and her/his family’s social conditions are strong predictors of cognitive ability in childhood. Interventions for promoting a healthy early childhood development are needed targeting children at risk of low IQ so that they can reach their full cognitive potential.

**Electronic supplementary material:**

The online version of this article (doi:10.1186/s12887-014-0308-1) contains supplementary material, which is available to authorized users.

## Background

The level of intelligence of a child is an important determinant of health outcomes and quality of life in adulthood [1,2] and is regarded as an indicator of human capital [3]. The intrauterine period and the first two years of life are sensitive periods for cognitive function [4] because it is when key processes of brain development take place [5]. Exposure to risk factors during these early stages of life has a significant impact on the life cycle [6,7].

Cognitive ability is genetically and environmentally determined. Although about 50% of intelligence variation among individuals is attributed to genetic factors [8], evidence shows that cognitive ability is also shaped by environmental and social factors [9] that can be effectively addressed with early life interventions [10,11].

Yet, most evidence comes from high-income countries [12,13]. Determinants of cognitive ability may vary in low- and middle-income countries possibly due to different distributions of risk factors and confounders as well as distinct associations between exposures and outcomes [14]. For example, breastfeeding is more prevalent among well-off educated families in high-income countries while the opposite scenario is more common in low- and middle-income countries [15]. In addition, unfavorable socioeconomic conditions are main predictors of low cognitive performance [12,16,17] and socially determined lower intelligence quotient (IQ) rates may be much higher in low-income countries due to prevailing poor social conditions and inequalities [18,19].

In 2007, it was estimated that around 200 million children under 5 in low- and middle-income countries fail to reach their potential in cognitive development during childhood and adolescence [20]. These children are not developing to their full potential, which can contribute to the intergenerational transmission of poverty.

Health providers rely on scant evidence to identify subgroups of preschool children at risk of low cognitive performance. A predictive modeling analysis can be a valuable approach to identify early life risk factors affecting cognitive ability and can help give priority to children at risk who could benefit from advice and early interventions.

Data from the 2004 Pelotas Birth Cohort provide a great opportunity to assess the impact of prenatal and early childhood variables on cognitive ability of children. The present study aimed to identify early life determinants of low IQ at age 6 using a predictive modeling approach.

## Methods

A population-based birth cohort study started in the city of Pelotas, southern Brazil, in 2004. All hospital births throughout that year were identified during daily visits to the city’s five maternity hospitals (over 99% of deliveries take place in hospitals). There were recruited 4,231 live births of mothers living in the urban area of Pelotas, accounting for 99.2% of all births in urban population in 2004.

Mothers were interviewed and their children examined within the first 24 hours after birth. A structured questionnaire was administered to collect information on demographic, socioeconomic, biological and behavioral characteristics. Gestational age was estimated by the best obstetric estimate using the National Center for Health Statistics (NCHS) algorithm [21] from the last menstrual period when available and consistent with standard birth weight, height and head circumference growth curves for each week of gestational age [22]. When the date of the last menstrual period was unknown or inconsistent, the Dubowitz method [23] was used to provide clinical estimates of the maturity of newborn infants.

Children were evaluated in the perinatal period and followed up at mean ages of 3.0 (standard deviation [SD] 0.1); 11.9 (SD 0.2); 23.9 (SD 0.4); 49.5 (SD 1.7) and 81.0 (SD 2.7) months, with follow-up rates of 95.7%, 94.3%, 93.5%, 92.0% and 90.2%, respectively. Anthropometric measurements including height, and head, chest and abdominal circumferences were taken. A detailed description of the 2004 Pelotas Birth Cohort methods has been published elsewhere [24,25].

This study was based on information collected in the perinatal period and at 3 months, 12 months and 6 years of age. The follow-up at age 6 years was conducted from October 2010 to August 2011. Participants were evaluated at the study clinic and those who did not attend the scheduled visit at the clinic were evaluated at home. The evaluation visit at the clinic lasted about 3 hours and the psychological assessment took around an hour to complete. Children with serious conditions that can be associated with very low IQ (e.g., severe mental retardation and cerebral palsy) were excluded. Participants with complete IQ test information at age 6 were included in the analysis.

The Wechsler Intelligence Scale for Children-III (WISC-III) validated for the Brazilian population [26] was applied to assess IQ in children at age six. It was composed of 4 subtests: 2 verbal (similarities and arithmetic) and 2 performance (block building and picture completion). A short-form version of the scale was used because of time constraints as a large number of children had to be evaluated. This version was developed by Kaufman [27] and showed a correlation above 0.90 with IQ measured by the full scale.

Score conversion tables for the U.S. population were used to calculate IQ scores from the subtests. IQ scores were converted into z-scores for the analysis. The study outcome was low IQ at age 6 defined as z-score < −1. This cutoff value was used instead of the traditional cutoff of 70 because the scores are from a different population tested in less controlled conditions than those of a clinic setting. Score tables for the Brazilian population were not used [26] because the ones available were created for broader age groups and an effect of age on child’s IQ has been described (data not shown).

Potential predictors were selected based on the literature data and easy collection in primary care settings. Information on the following variables was collected in the perinatal follow-up: total household income (categorized into monthly minimum wages; Brazil’s monthly minimum wage in 2004 was equivalent to $80); maternal education (full years of formal schooling at the child’s birth); maternal and paternal smoking during pregnancy; mother’s and father’s skin color (reported by the mother); child’s gender; teenage parents; mother with a partner; number of siblings; father’s employment status; intended pregnancy; maternal level of physical activity before and during pregnancy (reported by the mother); number of prenatal care visits; maternal hospitalization during pregnancy; type of delivery; prematurity; low birth weight; and health problems at birth.

The following variables were collected during the follow-up at 3 and 12 months: maternal smoking; number of persons per room living in the dwelling; child hospitalization; presence of maternal mental condition during the child’s first year of life (a score ≥8 in the Self-Report Questionnaire [SRQ-20] when the child was 3 months old, or a score ≥13 in the Edinburgh Postnatal Depression Scale [EPDS] when the child was 12 months old); duration of breastfeeding; duration of exclusive breastfeeding; father’s engagement in activities with the child in the preceding week (score estimated from the mother’s reports of the father spending time with the child feeding, diapering, bathing soothing during bedtime, playing, tending or strolling); childcare during the first year of life; maternal self-rated health; and maternal perception of the child’s health status. Weight-for-age, height-for-age, head circumference-for-age and weight-for-height measures were taken and assessed based on the World Health Organization growth chart [28]. Deficits were defined as a z-score < −2 SD at any of the three follow-ups (perinatal, 3 months and 12 months).

Several predictors studied are based on information from both the mother and the father (e.g. parental skin color, teenage parents). When a piece of information was not available about the father, we used information about the mother only.

All analyses were conducted using Stata v.12.1 (StataCorp. 2011. Stata Statistical Software: Release 12.1 College Station, TX: StataCorp LP). Descriptive analyses were used to determine the distribution of predictors and low IQ in the study sample. A logistic regression analysis with calculation of odds ratios (OR) and confidence intervals (95% CI) was performed as part of the unadjusted analysis to estimate the effect of each predictor on the outcome. A description of missing data was also included. To explore the effect of missing data on the estimates, the associations of potential predictors with low IQ were compared between the restricted sample—the one with complete data for predictors and outcome in the final model—and the maximum available sample used in the unadjusted analysis.

A multivariate analysis with predictive modeling was performed. Ordinal variables that were associated with increased odds of low IQ in the unadjusted analysis were included in the multivariate linear regression analysis. All potential predictors were concomitantly included in the multivariable logistic regression model, which was reduced using forward and backward stepwise selection taking into account the significance of the likelihood-ratio test (p ≤ 0.05 for inclusion and p > 0.051 for exclusion).

The predictors that were excluded were then manually re-entered into the final model to ensure that no major predictor was left out. The variables child’s age, interview setting and IQ test evaluator remained in the model while the modeling was applied to assess their potential effect on IQ test results and to provide a more realistic estimate of the effects of potential predictors on the outcome.

The discriminatory power of the final model was assessed by the area under the receiver operating characteristic curve (AUC) and its 95% CI [29]. Model calibration was assessed using the Hosmer-Lemeshow goodness-of-fit test [30]. Internal validation of the model was assessed using 500 iterations each of bootstrap method with same-size samples [31]. A final regression model was estimated for each sample and AUC calculated. Model optimism was then calculated as the difference between model performance in the bootstrap sample and the original dataset, and the final AUC value was set.

The predicted probability of low IQ for each participant was obtained from the final model. Subsequently, cutoff values for suspected low IQ were set taking into account the sensitivity, specificity, positive and negative predicted values, proportion of correctly classified as having low IQ and percentage of positives for all cutoffs in the cohort.

The 1993 Pelotas birth cohort study measured IQ from a subsample of their participants in 1997, when the children were aged 4 years [32]. IQ was measured using four subtests of the WPPSI [33] instrument adapted to Portuguese (Cunha J: Manual do WPPSI, administração e crédito dos testes. 1992, unpublished). A brief form of the test was used [34], which it is composed for two verbal subtests (comprehension and arithmetic) and two execution subtests (figure completion and construction with cubes). This was the best data source we found in terms of comparability to our study in order to carry out an external validation [35]. IQ was measured in 615 children using a different test, however this makes part of the Wechsler family. Children were aged 4 years, which was reasonably close to our children, aged 6 years. More importantly, all predictors used in our model were available, but one (mother’s perception of child’s health). We first fitted a model similar to our original predictive model to the 1993 Cohort sample and then we calculated the calibration and discrimination of the model. Second, we used our proposed scoring in the 1993 Cohort sample and calculated sensitivity, specificity and predictive values. For this exercise, we added half of the points relative to the variable that was not available to the score of each child, so that the scoring could be comparable.

All 2004 Pelotas Birth Cohort follow-up waves were approved by the Federal University of Pelotas Medical School Research Ethics Committee. All mothers or guardians of the participating children signed an informed consent form before data collection.

## Results

Of 3721 cohort children assessed at the 6-year follow-up, 3533 had information available on IQ testing. Ten children with severe conditions were excluded from the analysis, totaling 3523 in the final sample. The number of missing values for each potential predictor ranged from 0 (childcare) to 186 (pre-natal visits). The amount of missing values was below 2% for most predictors studied (72%; 23 of 32). Most children (81.4%) were evaluated at the study clinic.

At age six, low IQ (z-score < −1) was detected in 16.9% (95% CI 15.6–18.1) of the children in the cohort. Table 1 shows a description of the sample according to potential demographic, socioeconomic and behavioral predictors. About one-fifth were children of non-white parents; most mothers were living with a partner (84%) and 83% of the fathers were employed at the time of their child’s birth. Almost half of the mothers were not employed during pregnancy and the child’s first year of life. Most families (53%) had a monthly income less than or equal to 2 monthly minimum wages. Fifteen percent of the mothers had 4 or fewer years of schooling; the number of persons per room was equal to or greater than 3 in 21% of the households and 11% of the children had 3 or more siblings. With respect to pregnancy-related behavioral variables, at least one parent smoked during pregnancy in 44% of cases, and 31% of the mothers reported smoking during the child’s first year of life.

**Table 1:** Description of potential demographic, socioeconomic, and behavioral predictors of low IQ and unadjusted associations*

**Characteristic**	**Rate n (%)**	**Low IQ n (%)**	**Unadjusted OR (95% CI)**
All	3523 (100)	594 (16.9)	
Mother’s and father’s skin color (n = 3518)			p = 0.0000
White mother and father or either one	2736 (77.8)	373 (13.6)	1
Non-white mother and father	782 (22.2)	219 (28.0)	2.5 (2.0–3.0)
Teenage parents (n = 3522)			p = 0.0007
Neither	2776 (78.8)	436 (15.7)	1
Both or either one	746 (21.2)	157 (21.1)	1.4 (1.2–1.8)
Mother with a partner (n = 3522)			p = 0.0069
No	556 (15.8)	116 (20.9)	1.4 (1.1–1.7)
Yes	2966 (84.2)	477 (16.1)	1
Father employed at the child’s birth (n = 3445)			p = 0.0000
No	590 (17.1)	155 (26.3)	2.1 (1.7–2.6)
Yes	2855 (82.9)	412 (14.4)	1
Mother employed between pregnancy and the child’s first 12 months of life (n = 3456)			p = 0.0000
No	1645 (47.6)	359 (21.8)	2.0 (1.7–2.4)
Employed either during pregnancy or the child’s first 12 months of life	1811 (52.4)	220 (12.2)	1
Household income at the child’s birth (n = 3522)			p = 0.0000
One or less than one monthly minimum wage	823 (23.4)	248 (30.1)	7.6 (5.3–11.1)
Up to 2 monthly minimum wages	1041 (29.6)	213 (20.5)	4.5 (3.1–6.6)
Up to 4 monthly minimum wages	1004 (28.5)	97 (9.7)	1.9 (1.3–2.8)
More than 4 monthly minimum wages	654 (18.6)	35 (5.4)	1
Maternal education (years of schooling) (n = 3490)			p = 0.0000
0–4	527 (15.1)	197 (37.4)	9.4 (7.1–12.4)
5–8	1458 (41.8)	306 (21.0)	4.2 (3.3–5.3)
9 or more	1505 (43.1)	90 (6.0)	1
Number of siblings at the child’s birth (n = 3522)			p = 0.0000
Two or less	3149 (89.4)	454 (14.4)	1
Three or more	373 (10.6)	139 (37.3)	3.5 (2.8–4.4)
Number of persons per room at age 12 months (n = 3423)			p = 0.0000
<3	1687 (49.3)	190 (11.3)	1
≥3	1736 (50.7)	384 (22.1)	2.2 (1.9–2.7)
Maternal level of physical activity during and after pregnancy (n = 3522)			p = 0.0000
Physically inactive	2844 (80.8)	535 (18.8)	2.5 (1.9–3.3)
Active either during or after pregnancy	678 (19.2)	58 (8.6)	1
Maternal and paternal smoking during pregnancy (n = 3522)			p = 0.0000
None	1982 (56.3)	242 (12.2)	1
At least one parent smoked	1540 (43.7)	351 (22.8)	2.1 (1.7–2.8)
Maternal smoking during the child’s first year of life (n = 3404)			p = 0.0000
No	2336 (68.6)	330 (14.1)	1
Smoked	1068 (31.4)	247 (23.1)	1.8 (1.5–2.2)
Number of father-child activities at age 12 months (n = 3424)			p = 0.0008
0–2	568 (16.6)	117 (20.6)	1.8 (1.3–2.5)
3–6	2288 (66.8)	387 (16.9)	1.4 (1.1–1.9)
7 activities**	568 (16.6)	70 (12.3)	1
Childcare during the first year of life (n = 3523)			p = 0.0008
No	3353 (95.2)	580 (17.3)	2.3 (1.3–4.1)
Yes	170 (4.8)	14 (8.2)	1

*Logistic regression analysis in children age 6. The 2004 Pelotas Birth Cohort Study.

**Score estimated from the mother’s reports of the father spending time with the child feeding, diapering, bathing soothing during bedtime, playing, tending or strolling.

CI = confidence intervals; OR = odds ratios; IQ = intelligence quotient.

Regarding biological and maternal and child health variables (Table 2), 16% of the mothers attended less than 6 prenatal visits and 11% were hospitalized during pregnancy. Prematurity, low birth weight and health problems at birth were reported in 13%, 9%, and 12%, respectively. About 40% of the children were breastfed for 12 months or more and only 8% were exclusively breastfed for 6 months. The rates of weight-for-age, height-for-age, head circumference-for age and weight-for-height deficits at any of the three follow-up assessments were 12%, 17%, 9%, and 5%, respectively. More than a third of the mothers (37%) had a favorable perception of their child’s health while 38% had a negative perception.

**Table 2:** Description of potential biological and health predictors of low IQ and unadjusted associations*

**Characteristic**	**Rate n (%)**	**Low IQ n (%)**	**Unadjusted OR (95% CI)**
All	3523 (100)	594 (16.9)	
Intended pregnancy (n = 3521)			p = 0.0000
Intended	1554 (44.1)	206 (13.3)	1
Unintended	1967 (55.9)	386 (19.6)	1.6 (1.3–1.9)
Prenatal care visits (n = 3337)			p = 0.0000
<6	541 (16.2)	158 (29.2)	2.5 (2.1–3.2)
≥6	2796 (83.8)	390 (14.0)	1
Maternal hospitalization during pregnancy (n = 3522)			p = 0.5425
No	3147 (89.3)	534 (17.0)	1
Yes	375 (10.7)	59 (15.7)	0.9 (0.7–1.2)
Maternal mental disorder during the child’s first year of life (n = 3375)			p = 0.0000
No	2189 (64.9)	296 (13.5)	1
Yes	1186 (35.1)	268 (22.6)	1.9 (1.6–2.2)
Type of delivery (n = 3522)			p = 0.0000
Vaginal	1920 (54.5)	392 (20.4)	1.8 (1.5–2.2)
Cesarean section	1602 (45.5)	201 (12.6)	1
Gestational age (n = 3521)			p = 0.0009
<37 weeks	470 (13.4)	105 (22.3)	1.5 (1.2–1.9)
≥37 weeks	3051 (86.6)	488 (16.0)	1
Birth weight (n = 3522)			p = 0.0003
<2500 g	304 (8.6)	75 (24.7)	1.7 (1.3–2.3)
≥2500 g	3218 (91.4)	518 (16.1)	1
Health condition at birth (n = 3514)			p = 0.0078
No	3111 (88.5)	503 (16.2)	1
Yes	403 (11.5)	87 (21.6)	1.4 (1.1–1.8)
Child’s gender (n = 3522)			p = 0.0003
Female	1701 (48.3)	246 (14.5)	1
Male	1821 (51.7)	347 (19.1)	1.4 (1.2–1.7)
Child hospitalization during the first year of life (n = 3424)			p = 0.0000
No	2801 (81.8)	434 (15.5)	1
Yes	623 (18.2)	140 (22.5)	1.6 (1.3–2.0)
Duration of breastfeeding (n = 3512)			p = 0.0000
<1 month	364 (10.4)	98 (26.9)	2.2 (1.7–2.9)
1–11 months	1792 (51.0)	298 (16.6)	1.2 (1.0–1.5)
≥12 months	1356 (38.6)	194 (14.3)	1
Duration of exclusive breastfeeding (n = 3474)			p = 0.0000
<1 month	1265 (36.4)	258 (20.4)	2.8 (1.8–4.3)
1–5 months	1899 (54.7)	301 (15.9)	2.1 (1.4–3.1)
≥6	310 (8.9)	26 (8.4)	1
Weight-for-age deficit during the first year of life (n = 3522)			p = 0.0000
No	3099 (88.0)	476 (15.4)	1
Yes	423 (12.0)	117 (27.7)	2.1 (1.7–2.7)
Height-for-age deficit during the first year of life (n = 3523)			p = 0.0000
No	2918 (82.8)	436 (14.9)	1
Yes	604 (17.2)	157 (26.0)	2.0 (1.6–2.5)
Head circumference-for-age deficit during the first year of life (n = 3522)			p = 0.0000
No	3211 (91.2)	497 (15.5)	1
Yes	311 (8.8)	96 (30.9)	2.4 (1.9–3.2)
Weight-for-height deficit during the first year of life (n = 3521)			p = 0.0634
No	3334 (94.7)	551 (16.5)	1
Yes	187 (5.3)	41 (21.9)	1.4 (1.0–2.0)
Mother’s self-rated health (n = 3421)			p = 0.0000
Excellent/very good	1269 (37.1)	137 (10.8)	1
Good/fair/poor	2152 (62.9)	437 (20.3)	2.1 (1.7–2.6)
Maternal perception of the child’s health status (n = 3424)			p = 0.0000
Excellent/very good	2119 (61.9)	253 (11.9)	1
Good/fair/poor	1305 (38.1)	321 (24.6)	2.4 (2.0–2.9)

*Logistic regression analysis in children age 6. The 2004 Pelotas Birth Cohort Study.

CI = confidence intervals; OR = odds ratios; IQ = intelligence quotient.

Tables 1 and 2 show potential predictor variables for low IQ as well as the results of the unadjusted analysis. Low IQ was more common among children of non-white parents; with 3 or more siblings; born to unemployed fathers; born to parents with low household income and maternal education; born to mothers who attended less than 6 prenatal visits; with low birth weight; living with more than 3 persons per room in the dwelling; who were breastfed for less than a month; and with weight-for-age, height-for-age and head circumference-for-age deficits. In the unadjusted analysis, all potential predictors were associated with lower IQ (p < 0.05), except maternal hospitalization during pregnancy and weight-for-height deficit.

It was identified 594 children with low IQ in the cohort. Thirty-two potential predictors were evaluated, resulting in 19 events for each potential predictor. Table 3 shows low IQ predictors selected using the stepwise method and coefficients were used to assign weights to each predictor. This model included the variables child’s gender, parents’ skin color, number of siblings, mother’s and father’s employment status, household income, maternal education, number of persons per room, duration of breastfeeding, head circumference-for-age and height-for-age deficit, parental smoking during pregnancy, and maternal perception of the child’s health.

**Table 3:** **Final adjusted logistic regression model including early life predictors of low IQ at age 6**

**Predictor**	**OR** ^**a**^ **(95% CI)**	**p-value**	**Weights**
Male	1.5 (1.2–1.8)	p = 0.0002	15
Skin color: both mother and father non-white	1.9 (1.5–2.1)	p = 0.0000	25
Father unemployed at the child’s birth	1.6 (1.2–2.0)	p = 0.0002	18
Mother unemployed during the child’s first 12 months of life	1.5 (1.2–1.8)	p = 0.0003	15
Household income at the child’s birth^b^	1.3 (1.2–1.5)	p = 0.0000	12^b^
Maternal education^b^	1.8 (1.6–2.2)	p = 0.0000	23^b^
Number of siblings at the child’s birth: 3 or more	1.8 (1.3–2.3)	p = 0.0001	22
Number of persons per room at age 12 month: 3 or more	1.6 (1.3–2.0)	p = 0.0000	18
At least one smoking parent during pregnancy	1.3 (1.1–1.6)	p = 0.0145	10
Duration of breastfeeding		p = 0.0000	
<1 month	2.2 (1.6–3.1)		31
1–11 months	1.3 (1.0–1.6)		10
≥12 months	1		
Head circumference-for-age deficit during the first year of life	1.7 (1.2–2.4)	p = 0.0022	20
Height-for-age deficit during the first year of life	1.3 (1.0–1.7)	p = 0.0524	10
Maternal perception of the child’s health status (good/fair/poor)^c^	1.4 (1.2–1.8)	p = 0.0009	14

^a^Adjusted for child’s age, interview setting, IQ test evaluator.

^b^The effect indicates increased odds of low IQ by predictor category.

^c^The reference category is excellent/very good health.

CI = confidence intervals; IQ = intelligence quotient.

The 2004 Pelotas Birth Cohort (n = 3312).

**Figure 1:**
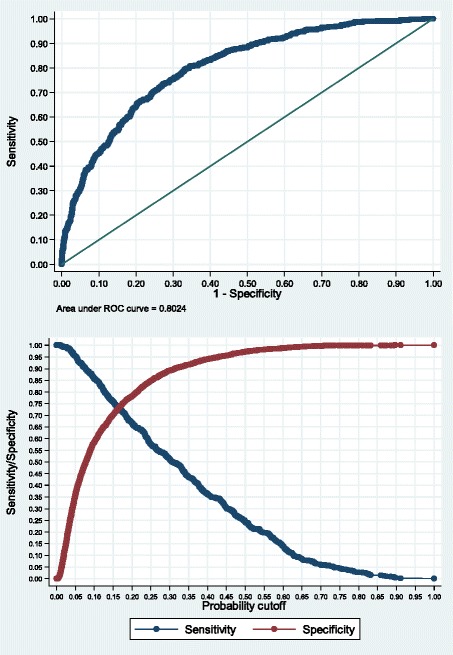
**Chart of AUC and probability of low IQ in children estimated from the final model.** AUC = area under the receiver operating characteristic curve.

Figure 1 shows an AUC for the final model of 0.80 (95% CI 0.79–0.82) indicating a good discriminatory power. Model optimism using internal validation techniques was 0.008 (95% CI 0.007–0.009) and the optimism-adjusted AUC was 0.79. The Hosmer-Lemeshow test showed a chi-square value of 1.84 (p = 0.9856), which indicates adequate model fit. It also shows sensitivity/specificity by the predicted probability of low IQ.

Table 4 presents 2 cutoff values of the predicted probability for suspected low IQ and test properties for the classification of children. A cutoff value of the probability that maximized sensitivity and specificity was 0.17, this corresponds to a cutoff value of >104 in the risk score for low IQ (sum of the weights of each predictor). Furthermore, another cutoff value with greater specificity was proposed in an attempt to reduce the proportion of false positives because of the low IQ rate found in this study (16.9%). An Excel including a table to calculate predictive scores for a given child is available upon request.

**Table 4:** Cutoff values of the probability for suspected low IQ*

**Test properties**	**Cutoff value**	
	**≥0.17**	**≥0.20**
Sensitivity	72.0%	66.5%
Specificity	73.6%	78.6%
Positive predictive value	35.0%	38.1%
Negative predictive value	93.0%	92.2%
Percentage of positives in the cohort	34.0%	28.9%
Correctly classified	73.3%	76.6%

*estimated from predictors in the final adjusted logistic regression model. Low IQ rate of 16.9% at age 6.

The 2004 Pelotas Birth Cohort (n = 3312).

As for the external validation in the 1993 Pelotas Cohort, we found a low IQ (z-score < −1) rate of 16.4% (95% CI 13.6–19.6) at age 4. The AUC for the model with all predictors was 0.75 (95% CI 0.71–0.79) and the chi-square value of the Hosmer-Lemeshow test was 3.69 (p = 0.8839). The cutoff of the risk score >104 showed a sensitivity of 70.3%, specificity of 68%, positive predictive value of 30.2%, negative predictive value of 92.1% and correctly classified of 68.4%.

## Discussion

This study identified the main early life predictors of low IQ at age six in children from a middle-income country birth cohort. The purpose was to identify predictors from the first year of life that can be routinely applied in clinical settings to screen children with suspected low cognitive performance who may benefit from advice or intervention at preschool age. Potential predictors were identified using a predictive model that showed good discriminatory power and adequate goodness of fit for the development dataset and for the external validation dataset.

The findings of this study on early predictors of low IQ are consistent with those reported in children from several countries with contexts different from the Brazilian one [9,19,36]. A full assessment of each association is outside the scope of this paper, but some aspects should be commented. Previous studies have reported lower IQ scores in male compared to female children [12,37,38]. Skin color is another characteristic that has been widely investigated. In general, poorer performance on IQ tests has been reported in non-white children [12,13,37,38].

In the Pelotas birth cohort, socioeconomic variables were strong key predictors of low IQ. Several studies that assessed the relationship between socioeconomic characteristics and cognitive ability found lower cognitive performance in children from families living in disadvantaged conditions including low income [12,17,37], unemployment [39,40], low education [13,41], large number of siblings [37,42] and crowded housing [38,43], compared to those better off. These associations were seen in many different age ranges and remained after adjusting for confounders. A possible explanation is that low socioeconomic condition is associated with several exposures that may negatively affect cognitive development such as poor nutrition, poor stimulation, and unfavorable family environment [18,44,45].

Another major finding of this study is the effect of growth, nutrition, and breastfeeding during the first year of life on cognitive ability. Children who were breastfed for a longer period were less likely to have low IQ than those who were not breastfed. It evidences a dose–response effect for this association, a finding that is similar to that reported in other studies [15,46,47]. In addition, children with no length and head circumference deficit from birth to the first year of life were less likely to have low IQ, which corroborates previous studies [48,49].

Other predictors of low IQ were smoking during pregnancy and maternal perception of the child’s health. There is an inverse relationship between smoking during pregnancy and cognitive ability of the child. Studies [50,51] are consistent with our finding that children exposed to smoking of either parent during pregnancy were at higher risk of low IQ than those non-exposed. Also, children of mothers with a poor perception of their child’s health were more likely to have low IQ, which is consistent with that reported by Bee [52].

Our results are also consistent with those reported in previous studies of the same cohort that investigated similar independent variables for an intelligence-related outcome at an earlier age [53-55]. They are also consistent with findings from more recent studies with other Brazilian populations and in high-income countries [56,57].

Maternal education, household income, parents’ skin color, duration of breastfeeding, head circumference and number of siblings were the most powerful predictors of low IQ at age six. Of a broad set of potential social and biological predictors explored those essentially social were the most impactful ones, which could mean that a high proportion of these children may require intervention.

Race-related health, wealth, education, and quality of life inequalities are prevalent in Brazil [58,59]. African descendants clearly have fewer opportunities, which is reflected in our results. The effects of parental skin color and the child’s gender should be interpreted as risk markers for low IQ rather than causal risk factors [60] because we only examined the predictive ability of these variables and did not assess whether there is a causal relationship between them. These risk markers for low IQ are valuable for screening population groups at higher risk of the outcome and identifying those children who would benefit from early interventions. In addition, these are markers of social risk containing the effect of unmeasured variables or variables measured with error, e.g., quality of life and access to public services. Besides, we should also bear in mind that dark-skinned children might receive less attention at school and/or experience discrimination in their own environment.

A strength of this study is its population-based birth cohort design that ensures temporal ordering of predictors and outcome and follows a large number of children from birth assessing a significant set of predictors and conditions using standardized anthropometrical and psychological assessment procedures. This is the first population-based study conducted in Brazil to assess early life determinants of low IQ in preschoolers with a predictive approach with internal and external validation. Another strength is the methods used for validating the risk score. For the internal validation, which assessed the model’s optimism based on resampling methods, we found a very low value for this score using the bootstrap approach, which is mainly due to a large sample size. For the external validation of the risk score, we found satisfactory results considering the differences (IQ test, age, etc.) which suggests that our proposed predicted model is robust. However, we should stress that the risk score is only applicable to the Brazilian context or similar contexts. We believe that in order to apply the risk score to other countries or contexts this should be adjusted locally.

The study has some limitations that need to be considered. First, there was a good amount of missing values on exposures and outcome, but a comparison of the results from the restricted sample and those of the unadjusted analysis showed the same direction and magnitude of associations [see Additional file 1]. Second, there may have been recall bias as information on some variables was reported by the mothers, but it was minimized by collecting data as early as possible. Third, there was no information available on maternal IQ, which is described in the literature as a major predictor of offspring IQ [17]. In the present analysis, maternal education was used as an approximate measure of maternal IQ.

The study found a major association of social determinants and poor performance on the IQ test right at the beginning of elementary school. This is a highly relevant finding. In the 2012 OECD Program for International Student Assessment (PISA) [61] that assessed mathematics, science and reading literacy among 15-year-old students and quality of education in 65 countries around the world, Brazil was among the 10-worst ranked countries in the 3 competence fields.

The practical implication of the study findings is that the risk of low cognitive ability in preschoolers can be predicted timely through a relatively simple routine assessment of early life social and biological variables in primary care settings. To identify at an early age those children with an increased risk of low IQ at age six will allow to referring them for appropriate advice and care.

A cutoff value of 17% of the probability of low IQ would result in 1 of every 3 children being suspected of low IQ, which could be problematic in terms of the absolute number of children requiring advice or intervention. However, there is a broad body of knowledge on easy, cost-effective interventions without any unfavorable effects [10,11,62-64] that do not require specialized equipment or staff and involve integrated family, school and health care actions for enhancing cognitive performance of children. These interventions can also have a positive impact on siblings, other children in the family and schoolmates.

Increased coordination across stimulation, nutrition, education and conditional cash transfer programs may help monitoring these children. Examples of integrated interventions and recommendations can be found in the literature [65-68]. In Brazil, there are many opportunities to integrate these interventions, such as the conditional cash transfer Bolsa Família program, which could require the recipient families to receive training in early childhood care and stimulation, particularly focusing on improving mother-child interaction. In this study, more than half of the families with children with low IQ (53.2%) were recipients of Bolsa Família program. Other intervention strategies could be implemented through the Family Health Strategy and the Community Health Agent Program.

## Conclusions

This study showed that a set of individual- and family-level biological and social predictors from the first year of life can predict, with good accuracy, low IQ at age six. Actions are needed to protect children against the negative impact of poverty, poor health and nutrition, and unfavorable family environment and to promote early childhood development so that they can reach their full social-emotional, physical and cognitive potential.

## Additional file

Additional file 1:
**Unadjusted associations of potential predictors with low IQ compared between the restricted sample and the maximum available sample.**

## Abbreviations

IQ: Intelligence quotient
WISC: Wechsler Intelligence Scale for Children
CI: Confidence intervals
SD: Standard deviation
OR: Odds ratios
AUC: Area under the receiver operating characteristic curve
