# Supplementary material for: Early life determinants of low IQ at age 6 in children from the 2004 Pelotas Birth Cohort: a predictive approach
Source: BMC Pediatr. 2014 Dec 16;14:308. doi: 10.1186/s12887-014-0308-1 (PMC4272809; doi:10.1186/s12887-014-0308-1)
Supplement: Additional file 1: — Unadjusted associations of potential predictors with low IQ compared between the restricted sample and the maximum available sample. [file 12887_2014_308_MOESM1_ESM.pdf]

# Early life determinants of low IQ at age 6 in children from the 2004 Pelotas Birth Cohort: a predictive approach

Authors: Fabio Alberto Camargo-Figuera, Aluísio J D Barros, Iná S Santos, Alicia Matijasevich, Fernando C Barros.

**Additional file 1**

**Additional Table 1. Unadjusted associations of potential predictors with low IQ compared between the restricted sample and the maximum available sample. The 2004 Pelotas Birth Cohort Study.**

| Characteristic | Maximum sample Unadjusted OR (95% CI) | Restricted sample Unadjusted OR (95% CI) |
|---|---|---|
| | | |
| Mother's and father's skin color (n=3518) | p = 0.0000 | p = 0.0000 |
| White mother and father or either one | 1 | 1 |
| Non-white mother and father | 2.5 (2.0–3.0) | 2.4 (2.0 – 2.9) |
| Teenage parents (n=3522) | p = 0.0007 | p = 0.0006 |
| Neither | 1 | 1 |
| Both or either one | 1.4 (1.2–1.8) | 1.5 (1.2 – 1.8) |
| Mother with a partner (n=3522) | p = 0.0069 | p = 0.1163 |
| No | 1.4 (1.1–1.7) | 1.2 (1.0 – 1.6) |
| Yes | 1 | 1 |
| Father employed at the child's birth (n=3445) | p = 0.0000 | p = 0.0000 |
| No | 2.1 (1.7–2.6) | 2.2 (1.7 – 2.7) |
| Yes | 1 | 1 |
| Mother employed between pregnancy and the child's first 12 months of life (n=3456) | p = 0.0000 | p = 0.0000 |
| No | 2.0 (1.7–2.4) | 2.0 (1.7 – 2.5) |
| Employed either during pregnancy or the child's first 12 months of life | 1 | 1 |
| Household income at the child's birth (n=3522) | p = 0.0000 | p = 0.0000 |
| One or less than one monthly minimum wage | 7.6 (5.3–11.1) | 7.4 (5.0 – 10.9) |
| Up to 2 monthly minimum wages | 4.5 (3.1–6.6) | 4.6 (3.1 – 6.7) |
| Up to 4 monthly minimum wages | 1.9 (1.3–2.8) | 1.9 (1.2 – 2.9) |
| More than 4 monthly minimum wages | 1 | 1 |
| Maternal education (years of schooling) (n=3490) | p = 0.0000 | p = 0.0000 |
| 0–4 | 9.4 (7.1–12.4) | 9.0 (6.8 – 12.0) |
| 5–8 | 4.2 (3.3–5.3) | 4.0 (3.1 – 5.1) |
| 9 or more | 1 | 1 |
| Number of siblings at the child's birth (n=3522) | p = 0.0000 | p = 0.0000 |
| Two or less | 1 | 1 |
| Three or more | 3.5 (2.8–4.4) | 3.5 (2.8 – 4.5) |
| Number of persons per room at age 12 months (n=3423) | p = 0.0000 | p = 0.0000 |
| <3 | 1 | 1 |
| ≥3 | 2.2 (1.9–2.7) | 2.2 (1.8 – 2.7) |
| Maternal level of physical activity during and after pregnancy (n=3522) | p = 0.0000 | p = 0.0000 |
| Physically inactive | 2.5 (1.9–3.3) | 2.4 (1.8 – 3.2) |
| Active either during or after pregnancy | 1 | 1 |
| Maternal and paternal smoking during pregnancy (n=3522) | p = 0.0000 | p = 0.0000 |
| None | 1 | 1 |
| At least one parent smoked | 2.1 (1.7–2.8) | 2.1 (1.8 – 2.6) |
| Maternal smoking during the child's first year of life (n=3404) | p = 0.0000 | p = 0.0000 |

| | | |
|---|---|---|
| No | 1 | 1 |
| Smoked | 1.8 (1.5–2.2) | 1.8 (1.5 – 2.1) |
| Number of father-child activities at age 12 months (n=3424) | p = 0.0008 | p = 0.0085 |
| 0–2 | 1.8 (1.3–2.5) | 1.6 (1.2 – 2.3) |
| 3–6 | 1.4 (1.1–1.9) | 1.4 (1.1 – 1.9) |
| 7 activities* | 1 | 1 |
| Childcare during the first year of life (n=3523) | p = 0.0008 | p = 0.0008 |
| No | 2.3 (1.3–4.1) | 2.6 (1.4 – 4.7) |
| Yes | 1 | 1 |
| Intended pregnancy (n=3521) | p = 0.0000 | p = 0.0000 |
| Intended | 1 | 1 |
| Unintended | 1.6 (1.3–1.9) | 1.6 (1.3 – 1.9) |
| Prenatal care visits (n=3337) | p = 0.0000 | p = 0.0000 |
| <6 | 2.5 (2.1–3.2) | 2.4 (1.9 – 3.1) |
| ≥6 | 1 | 1 |
| Maternal hospitalization during pregnancy (n=3522) | p = 0.5425 | p = 0.6891 |
| No | 1 | 1 |
| Yes | 0.9 (0.7–1.2) | 0.9 (0.7 – 1.3) |
| Maternal mental disorder during the child's first year of life (n=3375) | p = 0.0000 | p = 0.0000 |
| No | 1 | 1 |
| Yes | 1.9 (1.6–2.2) | 1.9 (1.5 – 2.3) |
| Type of delivery (n=3522) | p = 0.0000 | p = 0.0000 |
| Vaginal | 1.8 (1.5–2.2) | 1.6 (1.4 – 2.0) |
| Cesarean section | 1 | 1 |
| Gestational age (n=3521) | p = 0.0009 | p = 0.0023 |
| <37 weeks | 1.5 (1.2–1.9) | 1.5 (1.2 – 1.9) |
| ≥37 weeks | 1 | 1 |
| Birth weight (n=3522) | p = 0.0003 | p = 0.0002 |
| <2500 g | 1.7 (1.3–2.3) | 1.8 (1.3 – 2.3) |
| ≥2500 g | 1 | 1 |
| Health condition at birth (n=3514) | p = 0.0078 | p = 0.0033 |
| No | 1 | 1 |
| Yes | 1.4 (1.1–1.8) | 1.5 (1.2 – 2.0) |
| Child's gender (n=3522) | p = 0.0003 | p = 0.0006 |
| Female | 1 | 1 |
| Male | 1.4 (1.2–1.7) | 1.4 (1.1 – 1.7) |
| Child hospitalization during the first year of life (n=3424) | p = 0.0000 | p = 0.0001 |
| No | 1 | 1 |
| Yes | 1.6 (1.3–2.0) | 1.5 (1.2 – 1.9) |
| Duration of breastfeeding (n=3512) | p = 0.0000 | p = 0.0000 |
| <1 month | 2.2 (1.7–2.9) | 2.2 (1.7 – 2.9) |
| 1–11 months | 1.2 (1.0–1.5) | 1.2 (1.0 – 1.4) |
| ≥12 months | 1 | 1 |
| Duration of exclusive breastfeeding (n=3474) | p = 0.0000 | p = 0.0000 |
| <1 month | 2.8 (1.8–4.3) | 2.6 (1.7 – 4.0) |
| 1–5 months | 2.1 (1.4–3.1) | 2.0 (1.3 – 3.0) |
| ≥ 6 | 1 | 1 |

| | | |
|---|---|---|
| Weight-for-age deficit during the first year of life (n=3522) | p = 0.0000 | p = 0.0000 |
| No | 1 | 1 |
| Yes | 2.1 (1.7–2.7) | 2.1 (1.7 – 2.7) |
| Height-for-age deficit during the first year of life (n=3523) | p = 0.0000 | p = 0.0000 |
| No | 1 | 1 |
| Yes | 2.0 (1.6–2.5) | 2.0 (1.6 – 2.5) |
| Head circumference-for-age deficit during the first year of life (n=3522) | p = 0.0000 | p = 0.0000 |
| No | 1 | 1 |
| Yes | 2.4 (1.9–3.2) | 2.4 (1.8 – 3.1) |
| Weight-for-height deficit during the first year of life (n=3521) | p = 0.0634 | p = 0.1056 |
| No | 1 | 1 |
| Yes | 1.4 (1.0–2.0) | 1.4 (0.9 – 2.0) |
| Mother's self-rated health (n=3421) | p = 0.0000 | p = 0.0000 |
| Excellent/very good | 1 | 1 |
| Good/fair/poor | 2.1 (1.7–2.6) | 2.0 (1.6 – 2.5) |
| Maternal perception of the child's health status (n=3424) | p = 0.0000 | p = 0.0000 |
| Excellent/very good | 1 | 1 |
| Good/fair/poor | 2.4 (2.0–2.9) | 2.3 (1.9 – 2.8) |

CI = confidence intervals; OR = odds ratios; IQ = intelligence quotient
